# Hepatic Metastasis Revealing a Melanoma of the Penis: Case Report

**DOI:** 10.2196/37400

**Published:** 2022-09-27

**Authors:** Meriem Boui, Nabil Hammoune, Badr Slioui, Zakaria Zouaki, Mehdi Atmane, Abdelilah Mouhsine, Mohamed Amine Haouane, Mohamed Amine Azami, Mohamed Kaakoua, Ismail Assadi, Mohammed Boui, Ahmed Bouhamidi

**Affiliations:** 1 Department of Radiology Avicenne Military Hospital Cadi Ayyad University Marrakech Morocco; 2 Department of Anatomopathology Avicenne Military Hospital Cadi Ayyad University Marrakech Morocco; 3 Department of Oncology Avicenne Military Hospital Cadi Ayyad University Marrakech Morocco; 4 Department of Dermatology Military Hospital Mohammed V Medical University of Rabat Rabat Morocco; 5 Department of Dermatology Avicenne Military Hospital Cadi Ayyad University Marrakech Morocco

**Keywords:** melanoma, penis, immunotherapy, metastasis, lymph nodes, tumor, lesion, treatment, diagnosis

## Abstract

Melanoma of the penis is a rare tumor with a poor prognosis. We report the case of a 73-year-old patient with no significant medical history, admitted for deterioration of the general condition and bilateral inguinal lymph nodes. An abdominal ultrasound and thoraco-abdomino-pelvic CT (computed tomography) scan revealed metastatic liver nodules, the tumoral nature of which was confirmed by an anatomopathological examination. Further clinical examination revealed papular and ulcerated lesions of the penis located at the urethral meatus and glans penis. These lesions were biopsied and histologically assessed as melanoma. The contribution of imaging in penile tumors is generally not useful for diagnosis as clinical examination is key. However, it has its place in the assessment of locoregional and distant extension. In our case, it was the distant lesions that helped orient the diagnosis. The patient underwent immunotherapical treatment and is still alive 19 months after the diagnosis.

## Introduction

Melanoma of the penis is a rare tumor with a poor prognosis [1-3]. Since 1859, nearly 200 cases have been reported in the literature, representing less than 1.4% of primary penile carcinomas [1,4-6]. It is generally located on the glans penis (55%), followed by the foreskin (28%), the penile body (9%), and the urethral meatus (8%) [2,7]. Melanoma in situ of the penis is much rarer [8,9] and usually occurs in older adults [2]. Imaging does not usually have a role in diagnosis, but it does play a role in the workup.

We present a case of multifocal melanoma of the penis in a 73-year-old man with inguinal lymph nodes and hepatic nodules revealed via an abdominal ultrasound and thoraco-abdomino-pelvic CT (computed tomography) scan performed as part of the etiological investigation of an altered general condition.

### Case Report

The patient was 73 years old and had no previous history of illness. He presented with an altered general condition. The clinical examination revealed an altered patient with a World Health Organization performance status of 2, as well as visible and palpable bilateral inguinal adenopathy. The rest of the examination was unremarkable. The patient underwent a standard biological workup (complete blood count, liver workup, and renal workup), where no abnormality was found.

**Figure 1:**
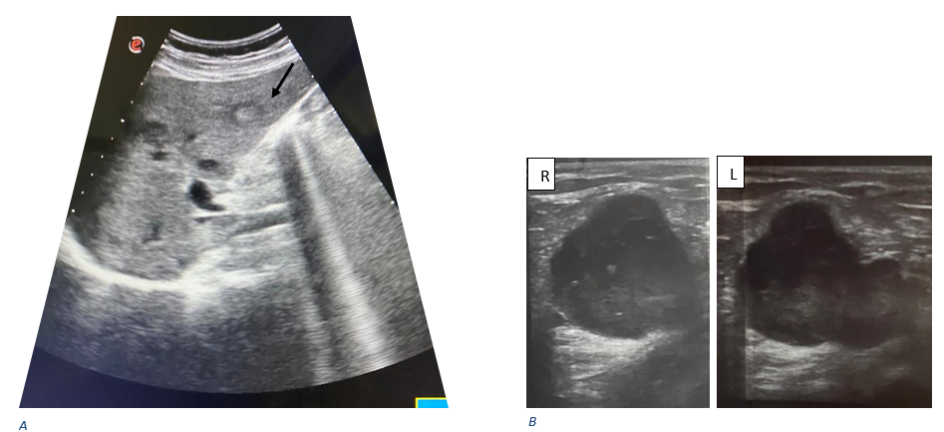
Abdominal ultrasound showing (A) hepatic lesions (black arrow) and (B) bilateral inguinal lymph nodes, right and left sides.

**Figure 2:**
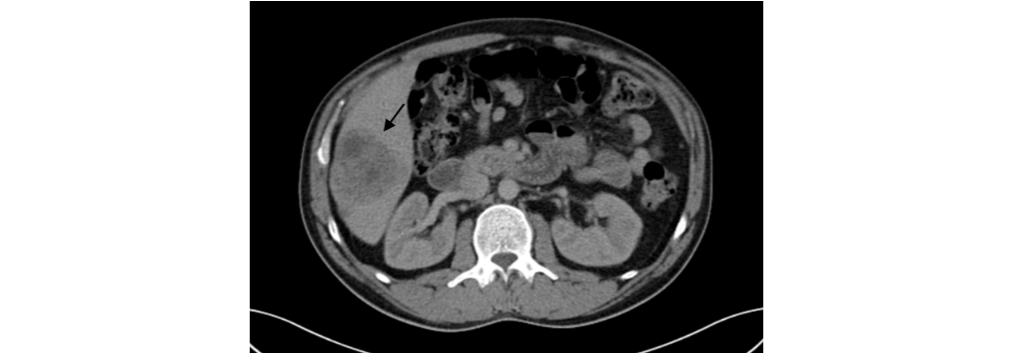
Thoraco-abdomino-pelvic CT scan showing hepatic lesions. CT: computed tomography.

He also underwent an abdominal ultrasound and a thoraco-abdomino-pelvic CT scan to look for a neoplastic cause. The latter showed secondary liver nodules (Figures 1 and 2) and multiple voluminous bilateral inguinal lymph nodes (Figure 2).

**Figure 3:**
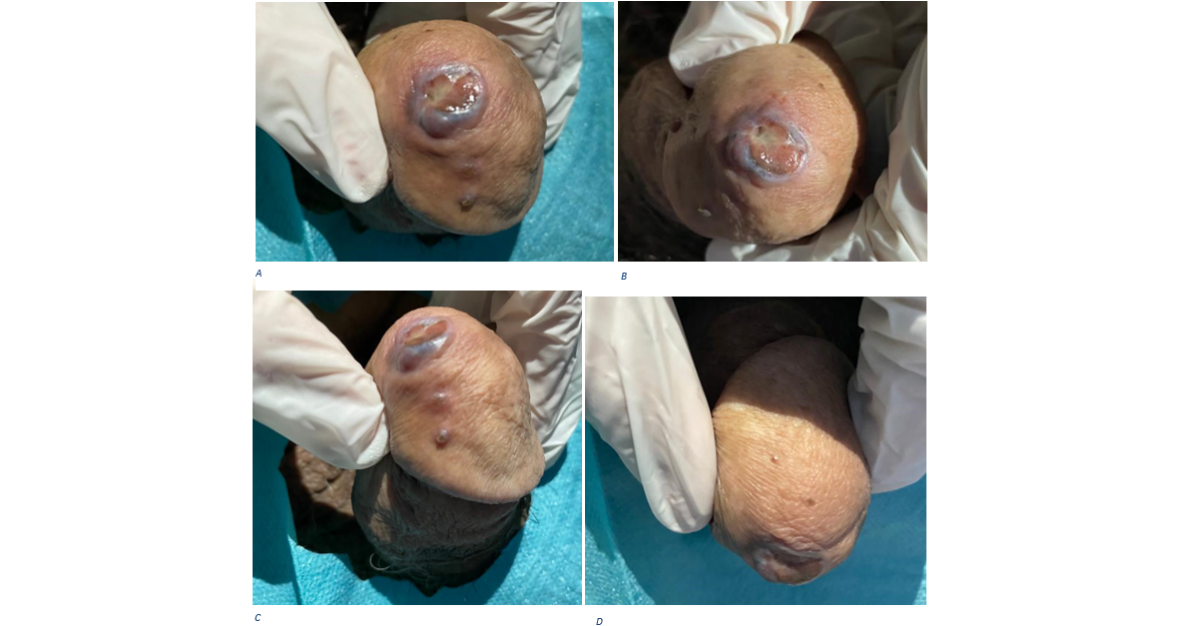
(A) and (B) Ulcerated lesion of the urethral meatus, (C) erythematous and brownish papules on the left side of the penis gland, and (D) satellite brownish papules on the dorsal surface of the penis gland.

**Figure 4:**
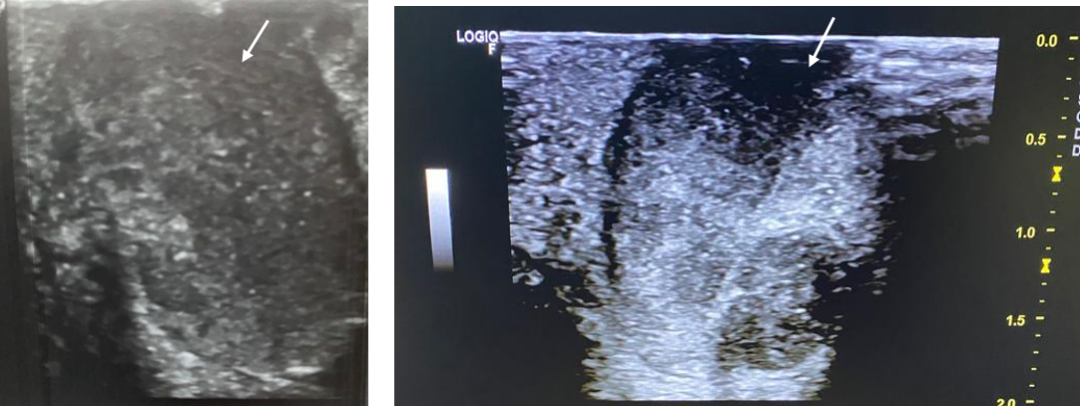
Ultrasound showing a penial hypoechoic and heterogeneous lesion.

An echo-guided biopsy of one of the hepatic nodules was performed and came back in favor of a metastatic nature. A second and more thorough clinical examination was performed and revealed, in addition to inguinal lymph nodes, an ulcerated lesion of the urethral meatus with a brown spot background associated with brownish papules in the vicinity of the glans penis (Figure 3). Ultrasound of the penial ulceration showed a hypoechoic and heterogeneous lesion (Figure 4). It should be noted that the patient is circumcised.

**Figure 5:**
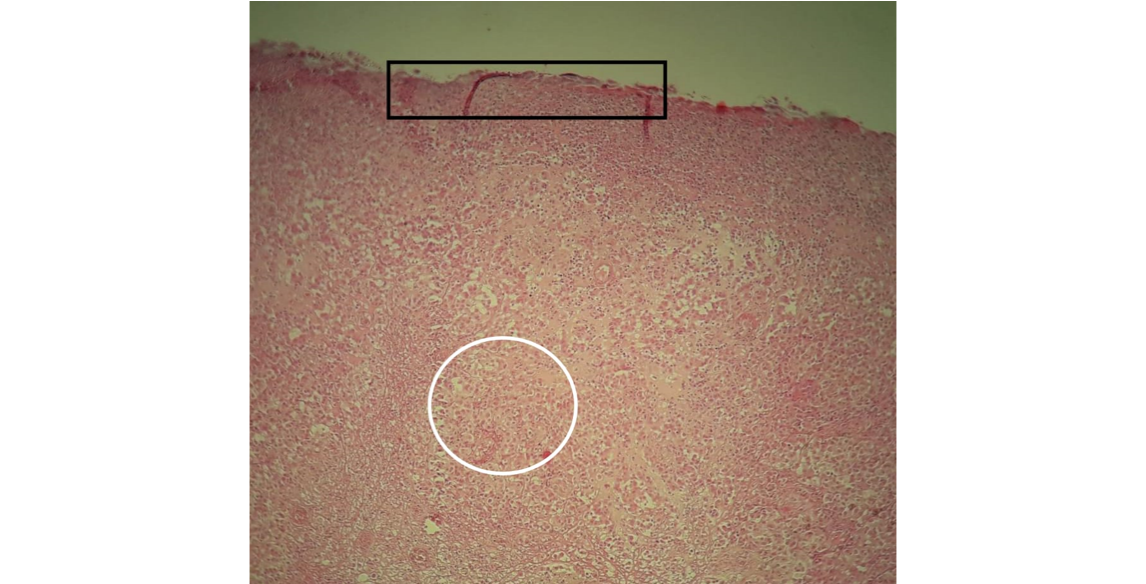
A histological image showing diffuse undifferentiated and ulcerated malignant tumor proliferation at ×40 magnification with hematoxylin and eosin staining (black rectangle: epithelium ulceration; white circle: tumoral proliferation).

**Figure 6:**
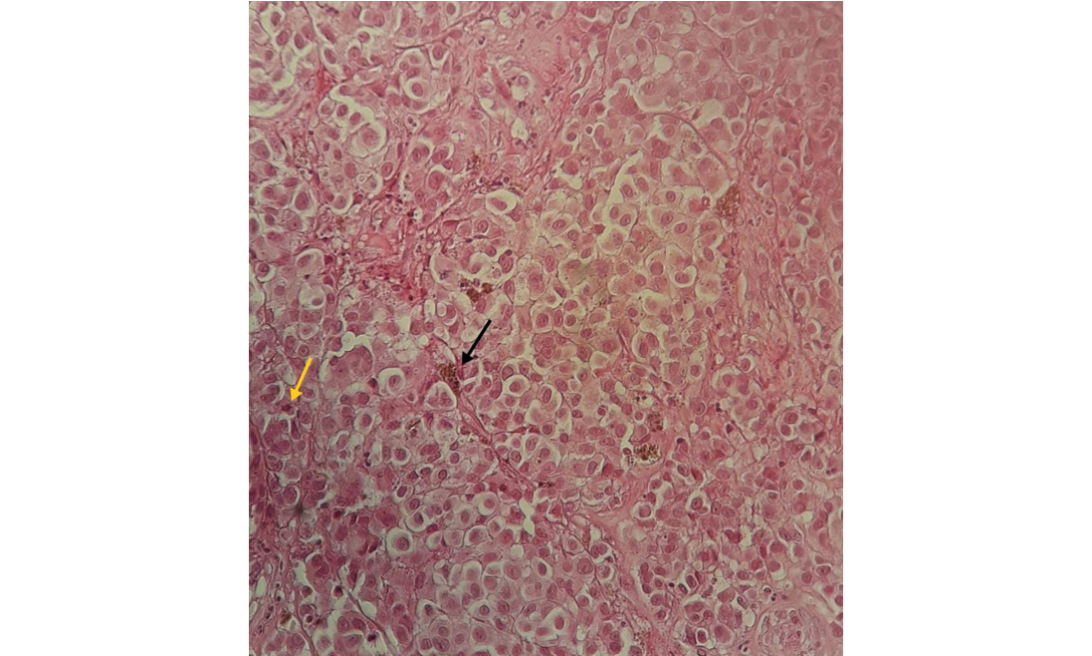
A histological image showing nests and lobules of highly nucleoted epithelioid malignant tumor cells at ×200 magnification with hematoxylin and eosin staining (yellow arrow: mitosis; black arrow: melanin pigment).

**Figure 7:**
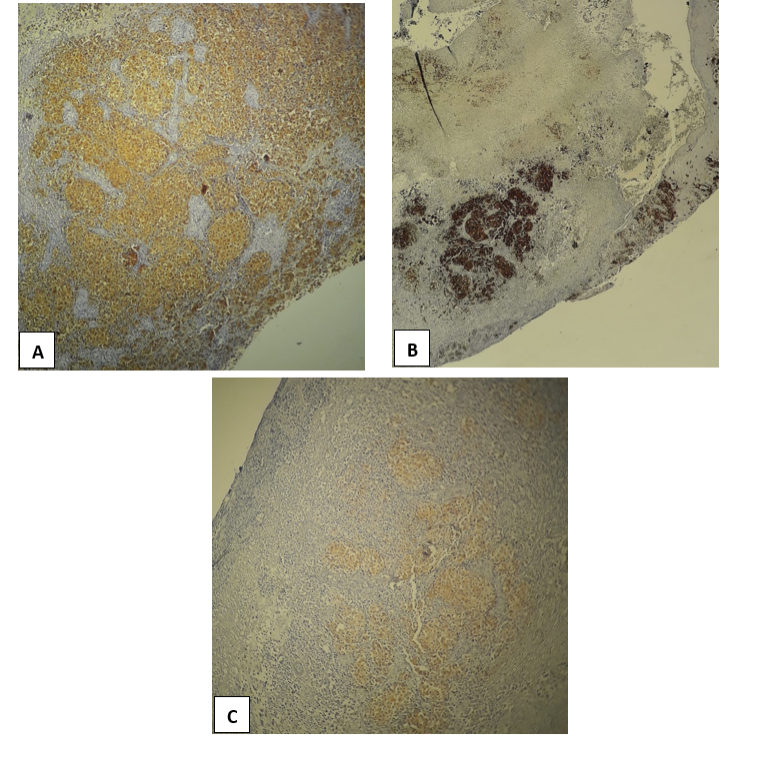
Immunohistochemical staining demonstrating staining with immunohistochemical melanoma markers (A) S100 protein, (B) HMB45, and (C) Melan-A.

A biopsy of the urethral meatus lesion was then performed, and the anatomopathological examination with the immunohistochemical profile was in favor of the melanoma type “not otherwise specified.” Indeed, it revealed a dermal lesion massively infiltrated by a malignant tumor proliferation, poorly limited epithelioma and rounded, with a clarified and abundant cytoplasm associated with foci of necrosis (Figures 5-7).

The proliferation infiltrated the surface epidermis. The Breslow index was estimated to be at least more than 4 mm with a level 4 on the Clark scale. After a multidisciplinary consultation meeting, surgery was not recommended because of hepatic and inguinal involvement and radical surgery would not have improved survival signiﬁcantly [1]. Subsequently, immunotherapy was recommended, and the patient was referred to the oncology department where he underwent immunotherapical treatment (pembrolizumab 200 mg/week). The patient is still alive 19 months after the diagnosis, and the last control CT scan showed stability of the hepatic lesion and a reduction of the inguinal nodes.

### Ethics Approval

The hospital’s ethics committee approved this study, and patient consent was obtained.

## Discussion

### Principal Findings

Melanoma of the penis is a rare lesion and has a poor prognosis [10]. It is a tumor that generally occurs in older adults, with the peak incidence occurring between 50 and 70 years of age [1]. The peak frequency of cutaneous melanoma at other body sites is between 40 and 49 years of age [10].

One of the major difficulties of penile melanoma is early diagnosis, which remains challenging because the initial clinical presentation of melanoma is often indistinguishable from a benign lesion. Melanoma can present as a brown, reddish-black, or bluish-pigmented lesion [10,11]. This is why any suspicious lesion should be biopsied early [10].

Various studies in the literature have reported melanomas of the penis and urethra [1,5,7,12,13]. In the latter case, the damage generally occurs in the fossa navicularis and more rarely in the pendulous, bulbous, and prostatic areas [1]. Only 5 cases of multifocal melanomas have been reported in the literature [1]. In our patient, melanomas were found in both the penis and urethral meatus.

There are no standard guidelines for the adequate staging of melanoma; however, most authors use a 3-stage system [14] to describe melanoma of the penis and the glans penis. Stage I refers to localized disease in the penis, stage II is melanoma involving the inguinal lymph nodes, and stage III refers to disseminated metastatic disease. Others use the American Joint Committee on Cancer (AJCC) tumor-node-metastasis (TNM) cancer staging system [15]. The patient presented in this case report was assessed as having AJCC stage III (T4 N2 M1) melanoma of the penis.

Imaging does not usually play an important role in the diagnosis but rather in the assessment of extension, particularly in the search for distant lesions, and in the follow-up.

The prognosis of penile melanoma is generally poor [10], most often because it is diagnosed late, especially at the stage of metastasis as in the case of our patient.

The prediction of the evolution of melanoma is based mainly on the tumor thickness. It had been proven that some factors worsen the prognosis such as tumor thickness >3.5 mm, presence of ulceration and microsatellites, and tumor diameter >15 mm [10]. This type of tumor has a poor prognosis and metastasizes rapidly. It presents very variable clinical manifestations from macules to papules and nodules, all of varying color. The survival rate at 2 years and 5 years is 63% and 31%, respectively [10].

Early diagnosis is important because of the high risk of distant metastases. On the other hand, if the tumor is diagnosed early, it is potentially curable [16]. However, in common practice, it is usually revealed at a late stage.

The lack of public prevention and the sensitivity of the melanoma site make early diagnosis difficult. Treatment is based on surgery when there is no distant extension [17]. The gold-standard treatment is based on resection of the lesion while preserving the organ [1,2]. The search for sentinel and inguinal adenopathy is essential, and a lymphadenectomy is sometimes necessary [1]. The prognosis remains poor due to the lack of effective chemotherapy.

### Conclusion

Malignant melanoma of the penis is a rare disease often associated with a high incidence of metastasis generally due to delayed diagnosis. The prognosis for survival is poor even when treated.

## Abbreviations

AJCC: American Joint Committee on Cancer
CT: computed tomography
TNM: tumor-node-metastasis
